# Sensitivity of brain MRI and neurological examination for detection of upper motor neurone degeneration in amyotrophic lateral sclerosis

**DOI:** 10.1136/jnnp-2021-327269

**Published:** 2021-10-18

**Authors:** Abram D Nitert, Harold HG Tan, Renée Walhout, Nienke L Knijnenburg, Michael A van Es, Jan H Veldink, Jeroen Hendrikse, Henk-Jan Westeneng, Leonard H van den Berg

**Affiliations:** 1 Department of Neurology, University Medical Centre Utrecht Brain Centre, Utrecht, The Netherlands; 2 Radiology, University Medical Center Utrecht, Utrecht, The Netherlands

**Keywords:** ALS, MRI, clinical neurology

## Abstract

**Objectives:**

To investigate sensitivity of brain MRI and neurological examination for detection of upper motor neuron (UMN) degeneration in patients with amyotrophic lateral sclerosis (ALS).

**Methods:**

We studied 192 patients with ALS and 314 controls longitudinally. All patients visited our centre twice and underwent full neurological examination and brain MRI. At each visit, we assessed UMN degeneration by measuring motor cortex thickness (CT) and pyramidal tract fibre density (FD) corresponding to five body regions (bulbar region and limbs). For each body region, we measured degree of clinical UMN and lower motor neuron (LMN) symptom burden using a validated scoring system.

**Results:**

We found deterioration over time of CT of motor regions (p≤0.0081) and progression of UMN signs of bulbar region and left arm (p≤0.04). FD was discriminative between controls and patients with moderate/severe UMN signs (all regions, p≤0.034), but did not change longitudinally. Higher clinical UMN burden correlated with reduced CT, but not lower FD, for the bulbar region (p=2.2×10^−10^) and legs (p≤0.025). In the arms, we found that severe LMN signs may reduce the detectability of UMN signs (p≤0.043). With MRI, UMN degeneration was detectable before UMN signs became clinically evident (CT: p=1.1×10^−10^, FD: p=6.3×10^−4^). Motor CT, but not FD, deteriorated more than UMN signs during the study period.

**Conclusions:**

Motor CT is a more sensitive measure of UMN degeneration than UMN signs. Motor CT and pyramidal tract FD are discriminative between patients and controls. Brain MRI can monitor UMN degeneration before signs become clinically evident. These findings promote MRI as a potential biomarker for UMN progression in clinical trials in ALS.

## Introduction

The heterogeneity in involvement of lower motor neurones (LMN) in spinal cord or brainstem, as well as upper motor neuron (UMN) and frontotemporal brain pathology, determines the clinical variability of patients with amyotrophic lateral sclerosis (ALS), and complicates early diagnosis and measurement of disease progression.^1^ In the clinic, LMN signs (eg, weakness), also supported by needle EMG findings, may be easier to detect and to quantify objectively in follow-up studies than UMN signs, which could become masked in limbs with significant LMN involvement.^1–3^ In clinical studies, such as trials to identify effective treatment for ALS, the Revised ALS Functional Rating Scale (ALSFRS-R) is used to measure functional decline.^4^ However, ALSFRS-R items are especially sensitive to LMN signs,^4–6^ implying that clinical trials may well fail to measure UMN treatment effects. Therefore, a biomarker for UMN degeneration could increase the sensitivity and power of clinical trials.^7 8^

Brain MRI measurements, such as motor cortex thickness (CT)^9–11^ and pyramidal tract fibre density,^12 13^ can be used to assess grey and white matter degeneration in ALS, whereas transcranial magnetic stimulation (TMS) provides in vivo insight into UMN functioning.^14 15^ Cross-sectional studies show a relation between UMN signs and grey^16 17^ and white matter^18 19^ degeneration; however, a longitudinal comparison of sensitivity of UMN signs and brain MRI has not previously been undertaken.

Aware of the possibilities of brain MRI serving as an UMN biomarker in clinical studies, we used longitudinal multimodal brain imaging and neurological examinations to study which measure is the more sensitive to UMN degeneration in ALS.

## Methods

### Participants

We recruited 678 subjects from a prospective, age-matched and gender-matched, population-based register in The Netherlands^20^ between November 2009 and March 2019. Patients with at least two follow-up visits were selected, excluding 136 patients who had only attended once. The revised El Escorial criteria were used to establish a diagnosis of ALS.^21^ We excluded 36 participants with orthopnoea, claustrophobia or structural brain abnormalities (ie, brain tumours, stroke), resulting in a study population of 506 (192 patients; 314 controls). Participants gave written informed consent.

### Data acquisition

All patients visited our clinic twice with an 4–6 month interval. At both visits, we performed a neurological examination of five body regions (bulbar region and limbs) for presence of UMN and LMN signs (figure 1.1). Healthy control participants were also examined neurologically, to be certain none of them had UMN signs that might confound the results. A standardised protocol, derived from the neurological examination in our motor neuron disease (MND) expertise centre, was used by each examiner (online supplemental table 2). Examiners were extensively trained before taking part in our study, even if they were already fully qualified. Examiners regularly take part in the MND outpatient clinic alongside their scientific work. During the 10-year study period a median of three examiners performed the neurological examination each year. In addition to neurological examination, we assessed clinical characteristics,^22^ functional status^5 23 24^ and cognition and behaviour status,^25^ and acquired a high-resolution T1-weighted and a diffusion-weighted image (DWI) of the brain using a 3T Philips (Philips Medical Systems, Best, the Netherlands) Achieva Medical scanner (figure 1.2). Acquisition parameters have been described in detail previously.^16^

10.1136/jnnp-2021-327269.supp1 Supplementary data

**Figure 1:**
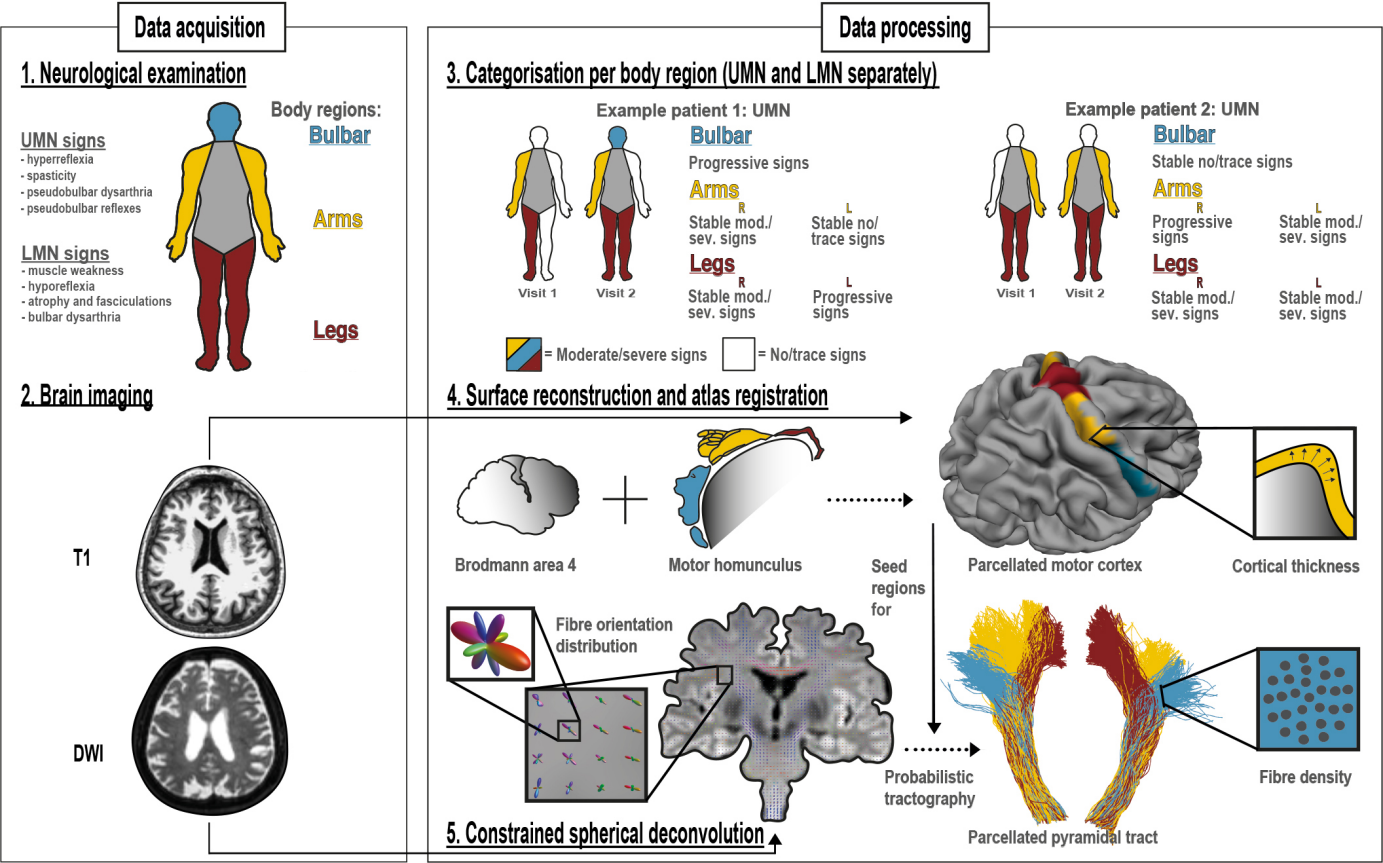
Study design. At each study visit, we acquired a detailed neurological examination (1) and structural and diffusion weighted imaging of the brain (2). Five body regions were examined (bulbar region and each limb) for UMN and LMN signs. Per body region, for UMN and LMN separately, we categorised patients as stable no/trace signs, progressive signs and stable moderate/severe signs (3). For the brain, we reconstructed surfaces and parcellated the primary motor cortex (Brodmann area 4) according to the motor homunculus. These motor cortex regions correspond to the body regions that were neurologically examined (4). From the DWI data, we calculated fibre orientation distributions applying constrained spherical deconvolution. with the motor cortex regions as seed regions, and the brain stem as target region, we reconstructed the pyramidal tract with a probabilistic tractography algorithm (5). Cortical thickness and fibre density per body region were used as measurements of UMN degeneration. All figures were created by the authors. DWI, diffusion-weighted imaging; LMN, lower motor neuron; mod, moderate; sev, severe; UMN, upper motor neuron.

### Data processing: neurological examination

To assess UMN and LMN involvement, we used the previously validated score by Devine *et al* (Devine score) which grades signs according to four scores: 0=no involvement, 1=trace involvement, 2=moderate involvement and 3=severe involvement.^26 27^ Based on these scores on two consecutive visits, we allocated patients to three categories according to involvement of five body regions: patients with no or trace UMN signs at both visits (stable no/trace UMN signs), patients with progression to moderate or severe involvement of UMN signs between visits (progressive UMN signs) and patients with moderate or severe UMN at both visits (stable moderate/severe UMN signs) (figure 1.3 and online supplemental table 3). Since the Devine score does not contain a score for the thoracic region, we did not include this body region in our analyses. UMN and LMN signs were uniformly documented by examiners and scored by the authors according to the Devine scoring method. With this method, we aimed to minimise inter-rater variability, although this cannot be fully ruled out because of the intrinsic subjective components of neurological examination.^28^

10.1136/jnnp-2021-327269.supp2 Supplementary data

### Data processing: brain MRI

We used motor CT and pyramidal tract fibre density as measures of UMN degeneration (ie, the lower the cortical thickness and fibre density, the greater the UMN degeneration). To acquire motor CT, we processed the T1-data using FreeSurfer software (V.6.0), automatically segmented the cerebral cortical mantle of the left and right hemisphere (LH and RH)^29^; parcellated the primary motor cortex defined by Brodmann Area 4^30^ and divided each hemisphere into 30 segments, gathering segments to the leg (segments 1–8, from medial to temporal), arm (segments 9–19) and bulbar region (segments 20–30), following the motor homunculus,^31^ together comprising the whole motor cortex (figure 1.4).

To acquire pyramidal tract fibre density, we registered the DWI data to the T1 data and used FSL (V.6.0) to correct for motion, eddy-current and susceptibility distortion. We used Constrained Spherical Deconvolution (CSD) from MRtrix3 (V.3.0) to estimate the Fibre Orientation Distribution of the pyramidal tract^32^ and used the earlier defined motor cortex regions as seed regions for a probabilistic tractography algorithm with the brainstem as target, a pyramidal tract atlas^33^ as an inclusion mask, and other cortical and subcortical volumes as exclusion masks. Application of exclusion masks was necessary to be able to track only pyramidal tract fibres. For example, we wanted to exclude corticobasal ganglia fibres, because these are outside the scope of this study. We successfully generated bulbar, arm and leg parts of the pyramidal tract for both hemispheres and used these to calculate an average fibre density (figure 1.5).

### Data analysis

We calculated statistics using R (V.3.6.1). For groupwise comparisons, we used a generalised linear model with age, sex and total brain volume as covariates. For covariate selection, we used a stepwise model selection calculation, with combined forward selection and backward elimination using the Akaike information criterion. We started the selection calculation using: age, sex, handedness, presence of *C9orf72* repeat length expansion and total brain volume. After one of the sensitivity analyses, we also included disease duration in the aforementioned generalised linear model as covariate for motor CT. We estimated correlation with Spearman’s rank correlation, compared demographic and clinical characteristics with Kruskal-Wallis test and used linear mixed models for longitudinal analyses. We used a linear model in a sensitivity analysis to compare cortical thickness patterns to study lateralisation of limb involvement. We considered p<0.05 as significant. Please see (online supplemental table 1) for the statistical formulae and R-packages.

10.1136/jnnp-2021-327269.supp3 Supplementary data

### Characterisation of neuroimaging and neurological examination markers

We characterised the effect of ALS on the brain UMN using both cross-sectional and longitudinal data, following the somatotopic organisation of the motor cortex and pyramidal tract. To do this, we explored motor CT at every part of the motor cortex comparing patients and controls. We divided both motor cortices, from medial to temporal, into 30 segments each, thus spanning the whole motor homunculus. We created profiles of cortical thickness of the motor cortices to evaluate whether thinning in patients is uniformly distributed along the length of the motor cortex. For fibre density of the pyramidal tracts, we compared each part of the pyramidal tract between patients and controls. In both analyses, we corrected for multiple testing using the Bonferroni method. To examine whether there is longitudinal decline in motor CT and fibre density, we estimated cortical thickness and fibre density at the first and second visit for every region of the motor cortex and pyramidal tract (statistical formulae used are presented in online supplemental table 1.5). In this analysis, we included all patients, irrespective of whether UMN signs were present or absent in body regions. We also characterised clinical decline of patients with ALS during the study, using the UMN and LMN Devine score for each body region (online supplemental table 1.5).

### Correlation of motor CT and fibre density with corresponding clinical UMN signs

We explored the correlation between brain UMN degeneration and clinical UMN signs. For each body region, we compared the degree of clinical UMN burden (Devine UMN score) with cortical thickness of corresponding motor cortex region and with fibre density of corresponding pyramidal tract area. Because there is no clear clinical lateralisation of bulbar signs in ALS,^1^ motor CT and fibre density of the bulbar region were averaged between hemispheres. In ALS, the presence of LMN signs can mask UMN signs.^2^ To test whether LMN masking was occurring in this cohort, we included the clinical LMN burden at each body region as an interaction effect with clinical UMN burden in the generalised linear model (online supplemental table 1.2).

### Comparing motor CT and fibre density against longitudinal development of UMN signs

We examined whether brain MRI reveals UMN degeneration before UMN signs become clinically evident, by comparing regional motor CT and pyramidal tract fibre density from brain MRI for the different categories at the first visit, based on UMN signs at first and second visits. We allocated UMN signs using Devine scores into three categories and compared with controls, as described before. We examined if UMN degeneration, averaged for all body regions, could be measured by brain MRI by pooling clinical UMN burden from all body regions (online supplemental table 1.5).

To further investigate how regional motor CT and fibre density differs for each category, we analysed these measurements for each body region separately and performed between-category comparisons (online supplemental table 1.1).

We performed three sensitivity analyses. In the first analysis of this study, described above, we examined whether cortical thickness differs within motor cortex regions. Therefore, for the first sensitivity analysis, we examined whether focality of thinning of a motor cortex region influences the between-category comparisons. The rationale for this is that we average cortical thickness of entire regions, although segments within the regions are not all affected equally as far as clinical signs are concerned (eg, tongue more than lips in the bulbar region),^34^ so that focal effects can be averaged out. To examine this, we used the thinnest segments of each region and repeated the between-category comparisons of cortical thickness. In the second sensitivity analysis, we examined whether differences in UMN degeneration between categories could be explained by differences in disease progression or disease duration. To test these alternative hypotheses, we added these variables as covariates to the generalised linear model (online supplemental table 1.1). In the third sensitivity analysis, we examined with a linear model (online supplemental table 1.6) whether lateralisation of limb involvement influenced motor CT (ie, if the motor CT corresponding to the side of the body with presence of UMN signs in both arm and leg at visit 1, is different from the thickness in patients with no UMN signs, in either arm and leg at that side, at either visit).

### Comparing motor CT, fibre density and clinical UMN signs as biomarkers for UMN degeneration

We examined which measurement is more sensitive for detection of longitudinal UMN degeneration: brain MRI or neurological examination. We compared deterioration of motor CT and fibre density with worsening of UMN signs over time. We estimated mean slope of MRI measures and UMN Devine score over time for each body region (online supplemental table 1.5). By comparing these slopes, we aim to identify which measurement worsens more over time, and is, therefore, better able to capture UMN degeneration.

For significant slopes calculated in aforementioned analysis, we estimated sample sizes (online supplemental table 1.6) needed to detect significant change in 14.5 months (median duration of clinical trials in ALS),^35^ for both brain MRI measurement and neurological examination.

## Results

### Demographic and clinical characteristics

Between patients and controls, age at first MRI, handedness and ECAS scores were comparable (table 1, p=0.65, p=0.86 and p>0.55). There were more women in the patient group (p=0.022); this was taken into account in the statistical analyses. Because of the short survival of patients with ALS compared with controls, the visit interval for patients with ALS was intentionally shorter than for controls (p<0.001). Total ALSFRS-R score declined significantly between the two visits (p=3×10^−27^).

**Table 1:** Demographic and clinical characteristics

	ALS	Controls
n	192	314
Sex=female	78 (40.6)	95 (30.3)
Age at first MRI, years	62.4 (53.7–68.4)	62.5 (54.7–68.5)
Age at onset, years	60.5 (52.6–66.0)	
Handedness=right	150 (78.1)	241 (76.8)
Visit interval, m	4.4 (3.6–5.5)	14.0 (10.8–17.7)
Site of disease onset		
Bulbar region	49 (25.5)	
Arms, right/left/both	78 (40.6), 30/29/19	
Legs, right/left/both	65 (33.9), 28/26/11	
Diagnostic delay, m	9.8 (5.8–18.0)	
Diagnosis to study interval, m	4.0 (2.3–6.2)	
Disease duration, m	14.4 (10.0–23.4)	
*C9orf72* repeat length expansion	16 (8.8)	
FVC at diagnosis, % of predicted	101.0 (90.8–111.3)	
King’s stage at visit 1, 1/2/3/4/missing	71/54/42/0/25	
MiToS stage at visit 1, 0/1/2/3/4/missing	163/4/1/0/0/24	
ECAS total score	113 (102–119)	114 (106–119)
ECAS, ALS-specific score	85 (76–89)	85 (78–88)
ECAS, ALS-non-specific score	30 (26–32)	30 (28–31)
bvFTD at visit 1	9 (4.7)	
ALSFRS-R score at visit 1	41 (38–44)	
Progression rate at visit 1	0.4 (0.2–0.6)	
Progression rate between visits 1 and 2	0.6 (0.3–1.1)	

Data are count (%) or median (25th quantile–75th quantile). Progression rate at visit 1 is defined as the decline in total ALSFRS-R-score per month from symptom onset until first visit. Progression rate between visits 1 and 2 is defined as the decline in total ALSFRS-R-score per month between visits. Presence of bvFTD is based on the Rascovsky criteria for behavioural FTD.^20^ All patients had two follow-up visits; for controls this was not obligatory; 145 controls completed the second visit.

ALS, amyotrophic lateral sclerosis; ALSFRS-R, Revised ALS Functional Rating Scale; bvFTD, behavioural frontal temporal dementia; ECAS, Edinburgh Cognitive and Behavioural ALS Screen; FVC, forced vital capacity; King's, King’s clinical staging system; MiToS, stage in Milano-Torino functional staging system.

### Characterisation of neuroimaging and neurological examination markers

**Figure 2:**
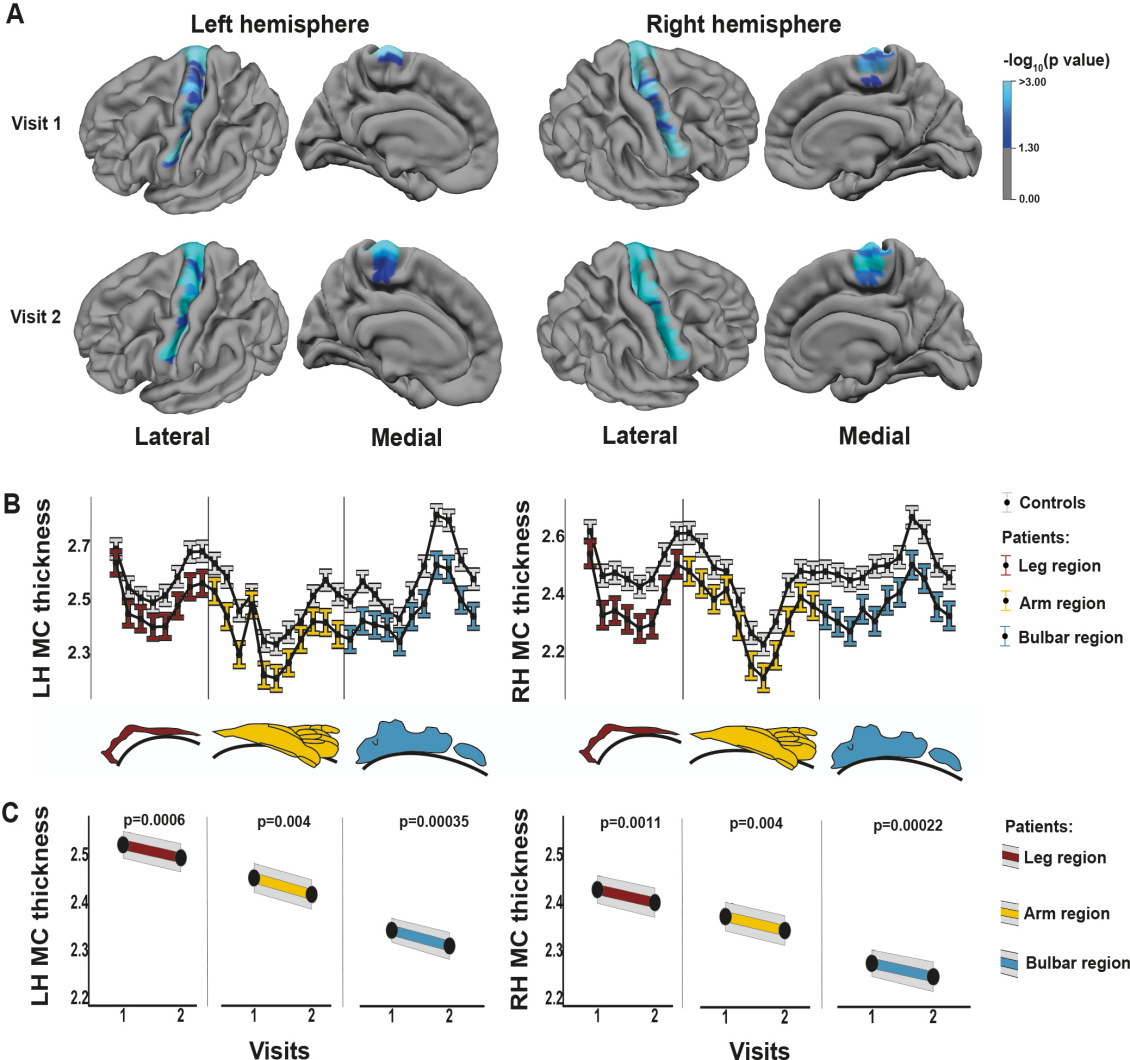
Patterns of motor cortex thinning. Motor cortex was divided into 30 segments per hemisphere and differences between controls (n=314) and patients with ALS (n=192) were measured (A, B). Segments with a significantly lower cortical thickness are shown in blue (p<0.05, Bonferroni corrected) (A). Thickness profiles of motor cortices of controls and patients are shown as estimated marginal means with error bars representing 95% CI (B). Colours indicate which segment belongs to the bulbar, arm and leg motor cortex region. For every region of the ALS motor cortex, longitudinal cortical thickness data are shown (C). All figures were created by the authors. ALS, amyotrophic lateral sclerosis; LH, left hemisphere; MC, motor cortex; RH, right hemisphere.

We found that all segments of the parcellated motor cortex of patients with ALS were thinner than in control participants at the first scan (figure 2A); statistical significance was reached in 52 of 60 segments. At the second scan, this difference was more pronounced and significant over all segments. The thinning of the motor cortex is most prominent in the RH leg region and in the bulbar region (figure 2B). Motor CT of all regions decreased over time (figure 2C). Between regions, we found, on average, no specific longitudinal thinning pattern for the total group of patients with ALS. For pyramidal tract fibre density, we found that, at both visits, patients differed from controls (bulbar pyramidal tract: LH p=0.015, RH p=0.011, arm pyramidal tract: LH p=0.0039, RH p=0.0015, leg pyramidal tract: LH p=0.00031). Difference in the RH leg pyramidal tract was not significant (p=0.079). Fibre density did not decrease over time for either controls or patients.

There was no difference in motor cortex thinning pattern for patients with a *C9orf72* repeat expansion compared with patients without this mutation. Controls did not show longitudinal motor cortex thinning.

**Figure 3:**
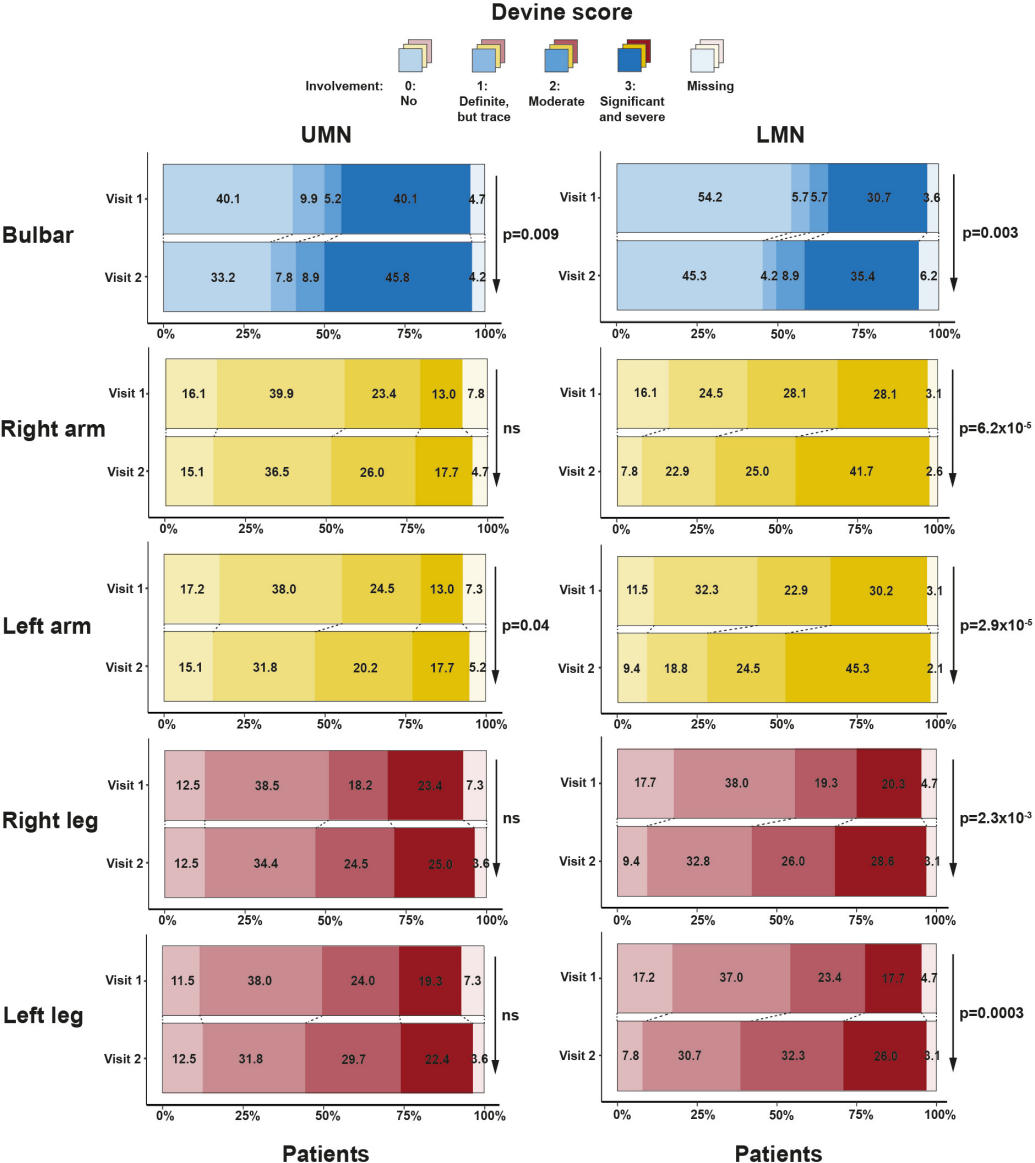
Clinical deterioration according to Devine score. For all body regions (bulbar and four limbs), we show percentages of patients in each Devine score at both visits. Both UMN and LMN scores are shown. We indicate significant change in Devine scores between two visits with arrow and p value. LMN, lower motor neuron; ns, not significant; UMN, upper motor neuron.

The Devine score of patients declined between two visits for both UMN and LMN scores (figure 3). For the UMN score, this was significant for the bulbar region and left arm, and for the LMN score, the decline was significant for all body regions.

### Correlation of motor CT and fibre density with corresponding clinical UMN signs

We found that a higher clinical UMN burden in the bulbar and both leg regions correlated with reduced motor CT in the bulbar region (r_s_=−0.46 p=2.2×10^−10^), right leg (r_s_=−0.17 p=0.025) and left leg (r_s_=−0.19 p=0.015), but not the arm regions (right: r_s_=−0.10 p=0.19, left: r_s_=−0.13 p=0.082). We found no significant correlation between the degree of clinical UMN burden and pyramidal tract fibre density. Higher LMN burden (Devine score 3) was associated with lower UMN burden (Devine score 0 or 1) corresponding to cortical thickness of the bulbar (p=0.0032), arms (left: p=0.043, right: p=0.033) and left leg regions (p=0.026). The same interaction effect was found for fibre density corresponding to the right arm (p=0.038).

### Comparing motor CT and fibre density against longitudinal development of UMN signs

**Figure 4:**
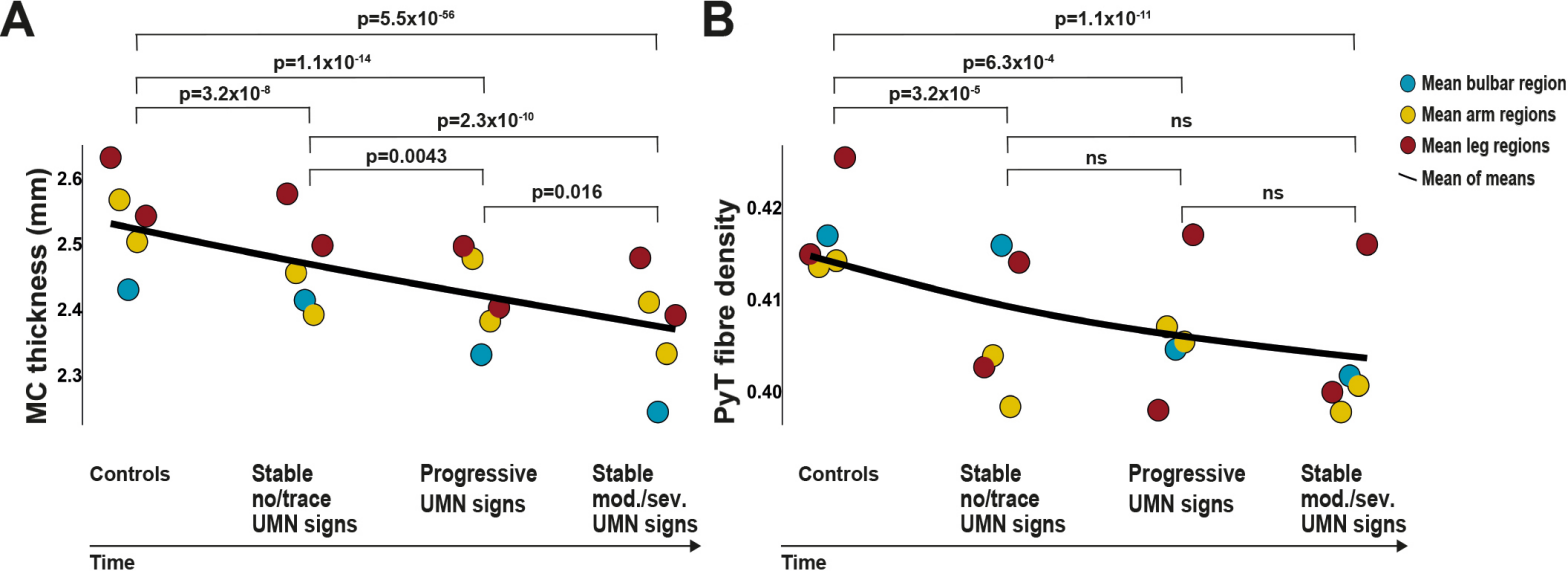
Mean UMN degeneration over time, from a healthy stage to severe UMN signs in a body region. Means of motor cortex thickness (A) and pyramidal tract fibre density (B) for all body regions were aggregated in this figure and a black line was drawn representing the mean of these means. This figure represents the average UMN degeneration from a healthy stage to severe UMN signs in a body region. From left to right, first datapoints represent the stage before onset of disease (data are from controls). The second stage shows the motor cortex thickness and fibre density of body regions that have no or trace UMN signs for 4.4 months (median visit interval). The third stage shows the motor cortex thickness and fibre density of body regions that have significant clinical progression of UMN signs between the first and second visits. The last stage shows motor cortex thickness and fibre density of body regions with moderate or severe UMN signs at time of scanning. We show p values for comparisons between these stages. MC, motor cortex; ns, not significant; PyT, pyramidal tract; UMN, upper motor neurone.

In this analysis, we examined whether brain MRI reveals UMN degeneration before UMN signs become clinically evident. Overall, we found that average UMN degeneration, as measured by motor CT and fibre density, worsened over subsequent categories (figure 4). For motor CT, every comparison was significant; for fibre density, every comparison with controls was significant (motor CT: p≤0.016, fibre density: p≤6.3×10^−4^).

**Figure 5:**
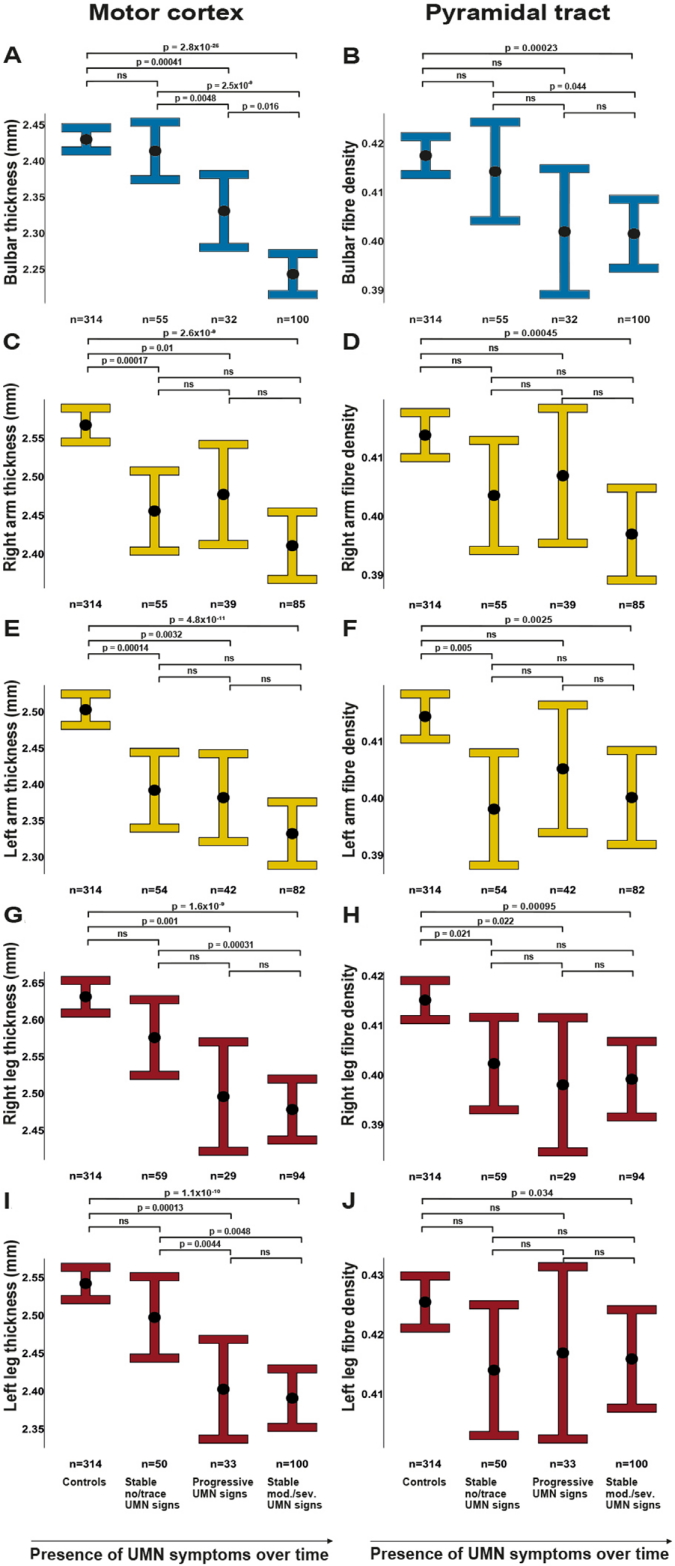
UMN degeneration for patients with and without clinically evident UMN signs. Between-category comparisons for all body regions (bulbar region and limbs) with estimated marginal means with 95% CIs of regional motor cortex thickness (A, C, E, G, I) and pyramidal tract fibre density (B, D, F, H, J) between controls, stable no/trace UMN signs patients (no or trace UMN signs at two visits), progression of UMN signs patients (UMN signs develop between first and second visit) and stable moderate/severe (mod./sev.) UMN signs patients (moderate or severe UMN signs since the first visit). The number of participants is shown for each group. Missing data for patients: bulbar region n=5, right arm n=13, left arm n=14, right leg n=16, left leg n=7. Comparisons of motor cortex thickness between patients are modelled with disease duration. ns, not significant; UMN, upper motor neurone.

When examining each body region separately (figure 5), we found that at the first visit, patients categorised as stable no/trace UMN signs had similar motor CT and fibre density to controls for all body regions, except for the cortical thickness corresponding to the arms and fibre density of the left arm.

At first visit, motor CT in these patients differed significantly from patients categorised as progression of UMN signs for the bulbar region and the left leg. Motor CT and fibre density in these patients differed significantly from patients categorised as stable moderate or severe UMN signs in the bulbar region, and also for motor CT in both legs.

Patients categorised as progression of UMN signs differed in motor CT from patients categorised as stable moderate or severe UMN signs in the bulbar region.

Patients categorised as stable moderate or severe UMN sign*s* differed from controls in both motor CT and fibre density in all five body regions.

In the first sensitivity analysis, we repeated the between-category comparisons with only the thinnest motor cortex segments. This did not change the differences in motor CT found in earlier analyses. In the second sensitivity analysis, we found no significant effect of disease progression on either motor CT or fibre density. We found that disease duration was associated with motor CT, but not with fibre density (CT: bulbar region: p=6.1×10^−4^, right arm: p=0.0032, left arm: p=5.9×10^−4^, right leg: p=1.2×10^−4^, left leg p=0.03). For this reason, we included disease duration in the generalised linear model as an additional covariate (online supplemental table 1.1). In the third sensitivity analysis, we found significant cortical thinning in the motor cortex corresponding to lateralised limb involvement of UMN signs (LH: p=2.6×10^−39^, RH: p=2.2×10^−17^, (online supplemental figure 1).

10.1136/jnnp-2021-327269.supp4 Supplementary data

### Comparing motor CT, fibre density and clinical UMN signs as biomarkers for UMN degeneration

**Figure 6:**
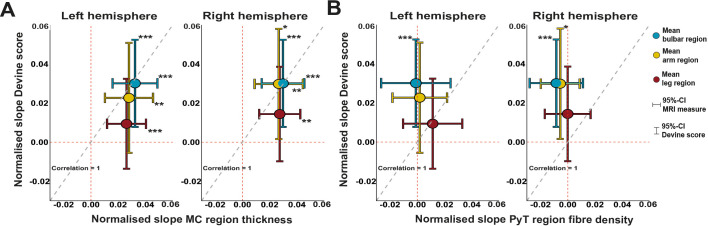
Sensitivity of brain MRI measurements compared with clinical UMN signs. Decline of MRI measurements (motor cortex thickness in panel (A), fibre density in panel (B) was compared with clinical decline in UMN Devine score between visits 1 and 2. The diagonal dashed grey line indicates equal sensitivity of clinical and MRI measurements. Means below the diagonal favour MRI measurements as most sensitive marker, while means above this diagonal favour the UMN Devine score as most sensitive marker for decline. 95% CIs are shown for all measures. 95% CI crossing the dashed red lines encompass the zero, indicating which regions and measures show a significant decline. Significant decline is indicated with asterisks, *p<0.05, **p<0.01, ***p< 0.001. MC, motor cortex; PyT, pyramidal tract; UMN, upper motor neurone.

We compared the progression of motor CT, fibre density and UMN signs over time and found that the normalised slope for motor CT is steeper than the slope of the UMN Devine score (figure 6), except for the left arm. Progressive thinning of motor cortex was found for all body regions (figure 6A, bulbar region: LH p=0.00035, RH p=0.00022, arms: LH p=0.004, RH p=0.004, legs: LH p=0.0006, RH p=0.0011). Progression of UMN signs was significant for the bulbar region and left arm (p=0.0009, p=0.036). For each body region, except the right leg, the normalised slope of clinical UMN signs was steeper than the slope for fibre density, which was significant for the bulbar region and left arm (figure 6B).

**Figure 7:**
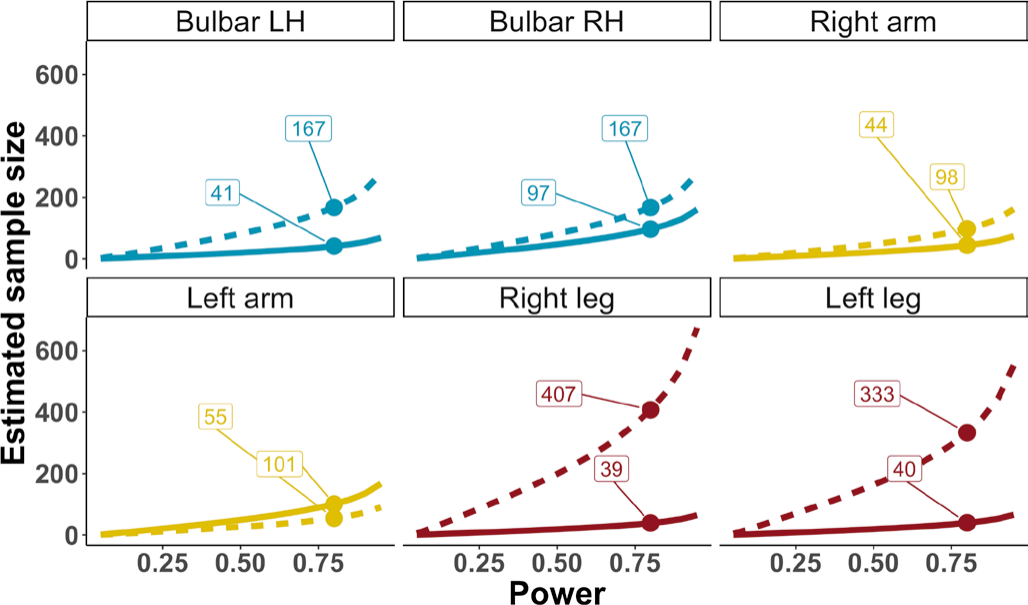
Sample size calculation for detection of significant UMN degeneration. Estimated sample sizes for detection of significant change in CT and UMN Devine score during a 14.5-month study period (the median duration of a clinical trial in ALS)^30^ at different probabilities to detect significant change (power). We highlight sample sizes at 80% power for UMN Devine score (dashed line) and CT (solid line). Data from figure 6 were used to calculate this figure. ALS, amyotrophic lateral sclerosis; LH, left hemisphere; RH, right hemisphere; UMN, upper motor neurone.

For motor CT and neurological examination, we estimated sample sizes and found that for motor CT, 41 patients, and for neurological examination, 167 patients are needed to detect significant change with a power of 80% in 14.5 months (medians for all body regions, figure 7).

## Discussion

In this study, we investigated UMN degeneration using longitudinal brain MRI and neurological examination in a large cohort of patients with ALS, and demonstrated that motor CT deteriorated faster during the study compared with clinical UMN signs. Therefore, we conclude that motor cortex neuroimaging parameters are more sensitive biomarkers for UMN involvement in ALS than clinical UMN signs. Clinical UMN signs correlated with motor CT in corresponding motor areas for bulbar and leg regions, but not for arms. This is most likely due to more severe LMN involvement in this region masking detectability of clinical UMN signs. When we pooled MRI data of all body regions, we found that these MRI markers of UMN degeneration precede clinical signs of UMN involvement. However, looking at individual body regions, there was heterogeneity and effects were larger for cortical thickness than fibre density. We propose that cortical thickness of motor cortex, longitudinally measured by brain MRI at relatively short intervals, may be a valuable outcome measure of clinical studies in ALS, such as therapy trials.

Previous studies have taken UMN signs of all body regions together and found a correlation with thinning of the whole motor cortex^16 36^ and white matter degeneration of the whole pyramidal tract.^19 37^ With the largest available longitudinal cohort to date, using multimodal imaging and a detailed neurological examination study protocol (n=192), we were able to examine each individual body region for UMN and LMN signs, in relation to their corresponding motor CT and fibre density region, by following the somatotopic organisation of the motor cortex and pyramidal tract.^33 34^ This approach may be more consistent with disease status and progression as found in clinical practice and clinical trials, as there are significant differences in functional decline and prognosis between bulbar onset and spinal-onset patients with ALS.^38^ In contrast to previous imaging studies that examined the brain MRI in relation to questionnaires on the neurological status or the site of onset of symptoms months to years before scanning,^10 16^ we used neurological examination as clinical measure of UMN signs, which may correspond better to underlying pathophysiology at the time of MRI scanning.^6 39^ This is further supported by the correlation we found between clinical UMN burden and UMN degeneration on MRI in the bulbar region and legs. However, we show that the exact clinical classification of UMN signs may be masked by loss of LMNs^2 3^ and we provide evidence that brain imaging is a more sensitive marker of UMN degeneration.^40^

Previous work examining the white matter part of the UMN primarily used diffusion tensor imaging (DTI), with measures such as fractional anisotropy. Traditional tensor metrics are less useful if a voxel has crossing fibres with a small fraction of the tract of interest (because fractional anisotropy is not fibre-specific), which is the case for the proximal part of the bulbar part of the pyramidal tract. For the pyramidal tract, this means that white matter integrity in the trajectory between the bulbar part of the motor cortex and internal capsule, where the superior longitudinal fasciculus crosses, can be better estimated using CSD.^12^ Another important advantage of CSD compared with DTI is that CSD has been shown to be capable of tracking arm and bulbar pyramidal tract (example shown in figure 1).^33^ In our study, we found more degeneration of motor CT than fibre density over time and clinical burden was not significantly correlated with lower fibre density. Longitudinal fibre density analysis of different pyramidal tract areas has not previously been reported in ALS. In this study, we show significant difference between controls and patients with moderate and severe UMN signs at time of scanning (figure 5), which underscores the discriminative value of fibre density of the pyramidal tract.^12 13^

Although we found a consistent overall pattern of UMN degeneration between categories in each body region, the largest effects were present in the bulbar region. One possible explanation is that at an unstratified level (figure 2), the bulbar region is the most atrophied region of the motor cortex.^10 16^ Moreover, for clinical reasons, we averaged the bulbar regions between hemispheres, probably resulting in less noisy estimates, compared with the estimates of a single hemisphere (the measurements of the limb regions). A possible underlying pathophysiological difference between bulbar and limb involvement is not known, although distinct disease effects have been recognised previously.^38 41^ The partial difference we found between cortical thickness and fibre density regarding correlation with clinical UMN burden might relate to the ongoing debate in the scientific field of ALS about the hypotheses of dying back and dying forward of upper and LMN and the white matter connections between them.^42^ This study does not aim to provide an answer in this debate.

There are potential limitations to our study. Attrition bias in our study may be due to rapid disease progression and development of orthopnoea. Because of a short median diagnostic delay and time from diagnosis to MRI scanning (9.8 months and 4 months, table 1), we were able to reduce this bias.^37 43^ Additionally, the clinical characteristics of this study population fall within the inclusion criteria of clinical trials in ALS.^44^ Moreover, with the protocol presented here, we found significant UMN degeneration within 4.4 months’ follow-up (table 1), well within the median duration of clinical trials.^35^ Whereas we examined the total length of the pyramidal tract, from pons to cortex, future studies might limit the region of interest of the pyramidal tract to the trajectory between pons and internal capsule, which might increase sensitivity for detecting longitudinal white matter changes, as degeneration is more pronounced here compared with regions closer to cortex.^16 45–47^ Longer follow-up duration might further increase sensitivity of white matter changes in the trajectory between pons and internal capsule, as some studies show white matter change at this location at later time points (>12 months). Future studies might also add direct comparisons of DTI and CSD longitudinally. Our study did not include other potential UMN biomarkers such as TMS^14 15^ or neurofilaments.^48 49^ In vivo assessment of UMN involvement is possible with TMS, although outcomes might be quite operator-dependent.^50^ Cerebrospinal fluid or plasma neurofilaments levels provide insight into whole brain degeneration, with the possible disadvantage of lacking specificity for UMN degeneration.^49^

Any statistical analysis of neurological examination data requires a categorisation strategy, with the disadvantage of losing the nuances of the neurological examination. Although this process is similar to the clinician’s task of interpreting the cumulated signs to reach a diagnosis, there is quite a consensus in the ALS community that we lack a good and reliable clinical scale for UMN involvement. As categorisation method, we considered using the Devine score and Penn UMN score^51^; both scales have been validated in patients with ALS and have adequate face validity from a clinician’s perspective. Since the Penn UMN score lacks LMN categorisation, which was fundamental to our study, we chose the Devine score. Direct comparison of Devine and Penn UMN scores with brain MRI to monitor UMN degeneration is open for future comparison. Future studies might also include more measurements in the same study period, to assess if the linear relationships we infer between two visits (ie, in figure 2C) are correct or if non-linear patterns should be considered. Our study does not yet provide evidence for using MRI data as diagnostic criteria, but the diagnostic and predictive value of MRI could be investigated in international, multicentre cohorts.^38^ Future studies might also examine generalisability of our findings to the ALS cohort in clinical trials as trials might tend to include more patients in advanced disease stages.^35^

Our study provides evidence that motor CT on brain MRI is a more sensitive measure of UMN degeneration and is detectable earlier in ALS than signs found on neurological examination: months before UMN signs became clinically evident, brain MRI revealed UMN degeneration. For clinical trials, these findings could provide an opportunity to improve measurement of therapeutic effects on UMN function. Current clinical trials most likely measure primarily LMN disease progression by using the ALSFRS-R.^4–6^ In the field of imaging in ALS, there are concerns of relatively small study sample sizes^6^ and mismatches between clinical and imaging metrics,^39^ which might withhold application of brain MRI as biomarker in clinical trials.^7^ Our study, however, revealed significant UMN degeneration over the course of 4.4 months in a group of 192 patients (table 1), whereas the median clinical trial in ALS has a duration of 14.5 months and includes 216 patients.^35^ In 14.5 months, with a power of 80%, significant UMN degeneration is detectable with a median of 41 patients using motor CT, 75.4% less than would be necessary using neurological examination. This is probably due to less subjective estimates of brain MRI compared with the human practice of neurological examination. It is well known by clinicians that there is a considerable variability in the appreciation of UMN signs among physicians,^28^ including among experienced ALS specialists. For example, what is considered as a brisk reflex by a neurologist may be considered as a normal reflex by another physician. Brain MRI does not have this inter-rater variability. However, this study does not aim to replace neurological examination with brain MRI in clinical practice, both measurements have different implications for diagnosis and management. Based on the present results, we propose that brain MRI, especially cortical thickness of motor cortex regions, could become a biomarker for UMN degeneration in clinical studies and trials in ALS.

## Data Availability

Data are available on reasonable request.
