# Supplementary material for: Sensitivity of brain MRI and neurological examination for detection of upper motor neurone degeneration in amyotrophic lateral sclerosis
Source: J Neurol Neurosurg Psychiatry. 2021 Oct 18;93(1):82–92. doi: 10.1136/jnnp-2021-327269 (PMC8685620; doi:10.1136/jnnp-2021-327269)
Supplement: Supplementary data [file jnnp-2021-327269supp001.pdf]

Supplemental table 2. Detailed content of neurological examination.

Body region	UMN	LMN
Bulbar	Pseudobulbar affect, primitive reflexes, speed of tongue movements, spastic dysarthria.	Flaccid dysarthria, tongue atrophy, tongue fasciculations, tongue muscle strength.
Arms	Increased arm tendon reflexes (biceps, triceps, radial)*, pathological reflexes (Hoffmann's sign, elevated/crossed trapezius, deltoid, pectoral), increased muscle tone over joints (shoulder, elbow, wrist).	Atrophy and fasciculations (pectoral, deltoid, biceps, triceps, forearm, first dorsal interosseous muscle, abductor digiti minimi muscle, thenar muscles), muscle strength (MRC-score deltoid, biceps, triceps, wrist extensors, wrist flexors, finger extensors, finger flexors, first dorsal interosseous, abductor digiti minimi, opponens pollicis muscle), decreased muscle tone over joints (shoulder, elbow, wrist), decreased/absent arm tendon reflexes (biceps, triceps, radial).
Thoracic	Superficial abdominal reflexes	Fasciculations, abdominal muscle strength

Legs	Increased leg tendon reflexes (patellar, adductor, ankle)*, pathological reflexes (Babinski's sign), increased muscle tone over joints (hip, ankle).	Atrophy and fasciculations (quadriceps, hamstring, ankle extensors, ankle flexors, extensor, foot), muscle strength (iliopsoas, quadriceps, hamstring, ankle extensors, ankle flexors, extensor hallucis longus), decreased muscle tone over joints (hip, ankle), decreased/absent leg tendon reflexes (patellar, adductor, ankle).

Supplemental table 2. Detailed content of neurological examination.

For each participant, standardized neurological examination was performed. Each examiner had undergone extensive training on how to implement the protocol, even if they were already fully qualified. This protocol has the utmost overlap with the items of the neurological examination performed in our MND expertise centre. Study examiners also regularly participate as physicians in the outpatient clinic. Each body region was scored on the basis of these items according to the Devine score, although we note that not all items appear in the Devine score (i.e., features of the thoracic region). * Asymmetry of tendon reflexes was assessed in relation to atrophy and weakness (i.e. if tendon reflexes in the arm were symmetrical, but the right arm was atrophied, this was considered to be UMN involvement of the left arm). Abbreviations: UMN = upper motor neurone, LMN = lower motor neurone.
