# Supplementary material for: Sensitivity of brain MRI and neurological examination for detection of upper motor neurone degeneration in amyotrophic lateral sclerosis
Source: J Neurol Neurosurg Psychiatry. 2021 Oct 18;93(1):82–92. doi: 10.1136/jnnp-2021-327269 (PMC8685620; doi:10.1136/jnnp-2021-327269)
Supplement: Supplementary data [file jnnp-2021-327269supp002.pdf]

Supplemental table 3. Representative clinical example of UMN categories based on neurological examination in one patient.

Patient	Body region	Visit 1	Visit 2	Category
Woman, 69y, first visit 3 months after diagnosis, no cognitive deficits, normal *C9orf72* repeat length, negative family history for MND.	Bulbar	No dysarthria, normal tongue movement, one primitive reflex	No dysarthria, normal tongue movement, two primitive reflexes	Stable no/trace UMN signs
	Right arm	Normal reflexes, normal muscle tone	Pathologically brisk biceps and triceps reflexes, normal muscle tone	Progressive UMN signs
	Left arm	Pathologically brisk biceps reflex, normal muscle tone	Pathologically brisk biceps and triceps reflexes, increased muscle tone.	Stable moderate/severe UMN signs
	Right leg	increased knee and ankle reflexes, spasticity	Pathologically brisk knee and ankle jerk reflexes, spasticity	Stable moderate/severe UMN signs
	Left leg	Pathologically brisk knee and ankle jerk reflexes, spasticity	Pathologically brisk knee and ankle jerk reflexes, spasticity	Stable moderate/severe UMN signs

Supplemental table 3. Representative clinical example of UMN categories based on neurological examination in one patient.

Categorisation of UMN signs per body region based on the neurological examination at two visits. The authors, not the examiner, defined which body region was in which category using the Devine score as a tool. For display purposes, we only show UMN categories here. LMN categories for this patient were: bulbar: progressive LMN signs, arm right: progressive LMN signs, arm left: stable moderate/severe LMN signs, both legs: stable moderate/severe LMN signs. Abbreviations: y = years, MND = motor neurone disease, UMN = upper motor neurone, LMN = lower motor neurone.
