# Supplementary material for: Sensitivity of brain MRI and neurological examination for detection of upper motor neurone degeneration in amyotrophic lateral sclerosis
Source: J Neurol Neurosurg Psychiatry. 2021 Oct 18;93(1):82–92. doi: 10.1136/jnnp-2021-327269 (PMC8685620; doi:10.1136/jnnp-2021-327269)
Supplement: Supplementary data [file jnnp-2021-327269supp003.pdf]

Supplemental table 1. Used statistical test, R-packages, and formulae.

	Statistical test	Function	R-package	Version	Formula
1.	Generalized linear model	glm()	stats	3.6.1	MRI measurement ~ UMN group + age + sex + total brain volume + disease duration<br><br>MRI measurement ~ (UMN group * LMN group) + age + sex + total brain volume + disease duration
2.	Step-wise model selection calculation	step()	stats	3.6.1	MRI measurement ~ group + age + sex + handedness + *C9orf72* RE + total brain volume
3.	Spearman's rank correlation	cor.test()	stats	3.6.1	~ MRI measurement + group
4.	Kruskal-Wallis test	kruskal.test()	stats	3.6.1	Demographic variable ~ group
5.	Linear mixed model	lmer()	lmerTest	3.1.0	ALSFRS-R-score ~ time between visits + (time between visits | ALS subject)<br><br>CT ~ age + sex + total brain volume + time between visits + (time between visits | ALS subject)

				Devine score ~ age + sex + follow-up visits + (follow-up visits | ALS subject)
				MRI measurement ~ category + age + sex + total brain volume + (1 | body region)
				scale(MRI measurement) [or] scale(Devine score) ~ age + sex + time between visits + (time between visits | ALS subject)
6. Linear model	lm()	stats	3.6.1	thickness ~ group + age + sex

Supplemental table 1. Used statistical test, R-packages, and formulae.

For reproducibility reasons we show statistical formulae, functions and R-package versions, in order of appearance in Methods. Abbreviations: *C9orf72* RE = *C9orf72* repeat length expansion, ALSFRS-R = revised ALS functional rating scale, NA = not applicable.
