# Supplementary figures and images for: Sensitivity of brain MRI and neurological examination for detection of upper motor neurone degeneration in amyotrophic lateral sclerosis
Source: J Neurol Neurosurg Psychiatry. 2021 Oct 18;93(1):82–92. doi: 10.1136/jnnp-2021-327269 (PMC8685620; doi:10.1136/jnnp-2021-327269)

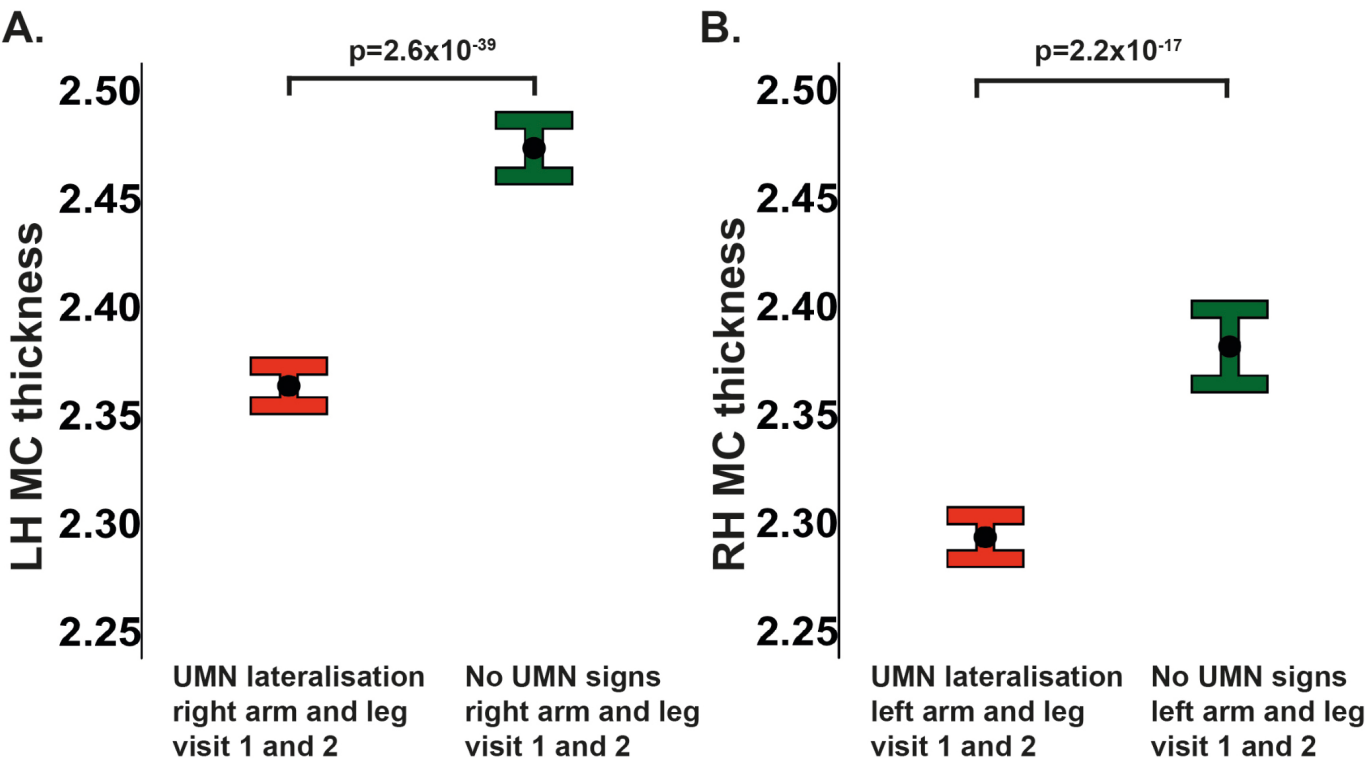

Supplement: Supplementary data [file jnnp-2021-327269supp004.pdf]
